# Preoperative investigation practices for elective surgical patients: clinical audit

**DOI:** 10.1186/s12871-024-02557-y

**Published:** 2024-05-23

**Authors:** Meseret Firde, Tikuneh Yetneberk

**Affiliations:** Department of Anesthesia, Debre Tabor University, Po.box: 272, Debre Tabor, Ethiopia

**Keywords:** Clinical audit, Preoperative test, Preoperative investigation, Pre-anesthesia workup

## Abstract

**Background:**

The findings of pre-operative investigations help to identify risk factors that may affect the course of surgery or post-operative recovery by contributing to informed consent conversations between the surgical team and the patient, as well as guiding surgical and anesthetic planning. Certainly, preoperative tests are valuable when they offer additional information beyond what can be gathered from a patient’s history and physical examination alone. Preoperative testing practices differ significantly among hospitals, and even within the same hospital, clinicians may have varying approaches to requesting tests. This study aimed to investigate preoperative testing practices and compare them with the latest guidelines from the National Institute for Health and Care Excellence (NICE).

**Methods:**

This three-month institutionally based study was carried out at the Debre Tabor Comprehensive Specialized Hospital from May 1 to July 30, 2023, including individuals aged 16 years and older who were not pregnant and had undergone elective surgery in the gynecological, orthopedic, and general units. Data on the sociodemographic characteristics, the existence of comorbidities, the invasiveness of surgery, and the tests taken into consideration by the guideline were gathered using a self-administered questionnaire. After rigorously analyzing and revising the results of preoperative investigation approaches, we compared them to the standard of recommendations. Moreover, the data was analyzed and graphically presented using Microsoft Excel 2013.

**Results:**

During the data collection period, 247 elective patients underwent general, orthopedic, and gynecological operations. The majority of patients, 107 (43.32%), were between the ages of 16 and 40 and had an American Society of Anesthesiologists (ASA) class one (92.71%). 350 investigations were requested in total. Of these, 71 (20.28%) tests were ordered without a justified reason or in contravention of NICE recommendations.

**Conclusions:**

In our hospital’s surgical clinical practice, unnecessary preoperative testing is still common, especially when it comes to organ function tests, electrocardiograms (ECGs), and complete blood counts (FBCs). When deciding whether preoperative studies are required, it is critical to consider aspects including a complete patient history, a physical examination, and the invasiveness of the surgery.

## Introduction

Preoperative investigations (PI) are tests performed prior to surgery to improve patient outcomes through planning, stratification, optimization, and perioperative management. The use of laboratory tests has long been an element of the preoperative evaluation of a patient’s fitness for anesthesia and surgery [1]. PI for patients scheduled for elective surgery typically includes a complete blood count (CBC), assessments of organ function, blood sugar level, urine analysis, chest radiography (CXR), and an electrocardiogram (ECG) [2].

Preoperative investigations can be categorized as either routine or indicated. According to the American Society of Anesthesiologists, routine preoperative tests are those performed without a specific clinical reason or purpose to detect a disease or disorder in a patient who shows no symptoms [3, 4]. Indicated tests are performed in response to a specific clinical need, such as confirming a diagnosis, evaluating the severity and advancement of a disease, or assessing the effectiveness of medication [5].

Preoperative tests are valuable when they offer information beyond what can be gathered solely from a patient’s medical history and physical examination [1]. Apart from assessing a patient’s suitability for surgery and anesthesia, PI plays a role in evaluating the advancement and severity of illnesses, anticipating postoperative complications, balancing the risks and advantages of surgery, and facilitating adjustments to the patient’s clinical treatment as necessary [5, 6]. Furthermore, PI can establish a baseline for future comparison, particularly when interpreting possibly abnormal postoperative test findings that may be challenging to understand independently [7, 8].

Preoperative testing practices exhibit significant variability across hospitals, and even within the same hospital, clinicians may adopt diverse approaches to PI requests. Despite their historical importance in pre-anesthetic assessment, numerous scientific studies in recent years have suggested that routinely ordering preoperative tests for generally healthy patients often offers limited value in identifying illnesses or influencing anesthetic care or outcomes [5, 10–12]. A significant portion of routinely conducted investigations, which can be expensive, may result in false-positive or borderline findings, prompting the need for further tests. This could impose psychological and financial difficulties, as well as delays or cancellations of surgery, potentially heightening the risks of morbidity and mortality [13, 14]. Furthermore, it can lead to inefficient resource allocation and an increased workload for laboratories, potentially compromising the quality of patient care.

Similarly, Dzankic et al. concluded that routine preoperative testing for hemoglobin, creatinine, glucose, and electrolytes may not be indicated in geriatric patients after conducting a prospective cohort study that evaluated the prevalence and predictive value of abnormal preoperative laboratory tests in 544 patients aged 70 years old undergoing non-cardiac surgery [15]. In a prospective study involving 400 patients that aimed to assess the effectiveness of preoperative investigations in identifying high-risk groups for complications, only 16% of the results showed abnormalities. However, of these abnormal results, only 0.013% prompted a change in perioperative management [16].

Instead, conducting a detailed patient medical history and physical examination is the most efficient way to detect abnormalities. Following this, only a few specifically chosen tests are administered, taking into account the patient’s condition, the complexity of the planned surgery, and the likelihood of blood loss [14, 17, 18]. A selected testing strategy reduces costs without sacrificing surgical safety or the quality of treatment [5].

Nowadays, there is a greater emphasis on low-value healthcare services [1]. Nowadays, when preoperative laboratory investigations are correlated with the patients’ histories and physical examinations, they are found to be beneficial and cost-effective, and there is a greater emphasis on low-value healthcare services [1, 8, 9, 19]. Several organizations, including the National Institute of Health and Clinical Excellence (NICE) and the Canadian Anesthesiologists’ Society (CAS), have issued guidelines to standardize testing and reduce costs [20]. The purpose of this audit was to identify disparities between the preoperative investigation procedures followed at Debre Tabor Comprehensive and Specialized Referral Hospital (DTCSH) and the latest preoperative investigation guidelines provided by NICE [20]. Secondly, our aim was to identify commonly ordered routine tests and assess the frequency of abnormal findings.

## Methodology

This three-month institutional-based study was carried out at the Debre Tabor Comprehensive Specialized Hospital from May 1 to July 30, 2023. The study included all patients older than 16 years who were not pregnant and were hospitalized for elective surgery under general or regional anesthesia in the orthopedic, gynecological, and general units during the study period. The study excluded patients scheduled for emergency surgeries, day-case surgery, cardiothoracic procedures, neurosurgery, and elective procedures under local anesthesia.

We used NICE recommendations as a standard for comparison since they are internationally accepted, clear, and easily applicable guidance, even in developing nations. By recommending which investigations to offer before minor, intermediate, and major surgery while taking particular comorbidities into account, this guideline addresses standard preoperative tests to reduce unnecessary testing. Likewise, the audit focused on the tests indicated in the recommendation, including resting echocardiogram, FBC, hemoglobin A1C (HbA1c) testing, hemostasis tests, and organ function tests.

A self-administered questionnaire that was constructed in compliance with the guidelines was used to collect the data. The nights before surgery, patients were asked about possible pregnancies and any undiscovered illnesses. On the day of surgery, at the end of the procedure, data pertaining to surgical invasiveness (major, intermediate, or minor) and PI (FBC, CXR, ECG, BUN, creatinine, electrolyte, coagulation profile, kidney and liver function test) was also gathered from the patient’s medical record and compared to the guideline for appropriateness. Aside from the preoperative investigations, the patient’s sociodemographic characteristics, such as age, gender, American Society of Anesthesiologists (ASA) status, and the presence and severity of comorbid illness, were recorded. It is preferred to collect data at the end of procedures to avoid missing tests that might be performed in the morning of surgery.

In addition, the results of each investigation were recorded after being assessed as normal or abnormal, depending on the laboratory report’s normal range. The data were securely collected by two masters of sciences degree (MSc) anesthetists who were not engaged in the clinical practice of preoperative investigation requests. The information gathered was inspected for completeness and accuracy. Moreover, the data was analyzed and graphically presented using Microsoft Excel 2013.

### Operational definitions

Minor surgery: surgical procedures including Excision of skin lesion, Myringotomy tubes, Hysteroscopy and Endoscopy/Colonoscopy [21].

Intermediate surgery: surgical procedures including hernia repair, laparoscopic, cholecystectomy, arthroscopy and tonsillectomy [21].

Major surgery: surgical procedures including total abdominal hysterectomy, endoscopic resection of the prostate, lumbar discectomy, thyroidectomy, total joint replacement, lung operations, colonic resection, and radical neck dissection [21].

Recommended (appropriate) investigation: the test that is recommended to be performed or considered after consideration of the particular comorbidities, sociodemographic profile, and types of surgery [21]:

Normal value ranges were defined using the institution’s laboratory ranges.

Preoperative laboratory testing: Preoperative investigation was defined for the purposes of this audit as any component of a laboratory or imaging test performed before surgery and considered by the guidelines.

## Results

The study included 247 patients who had elective surgery throughout the data collection period. The majority of patients, 107 (43.32%), were between the ages of 16 and 40 and had an American Society of Anesthesiologists (ASA) class one score of 229 (92.71%). Cardiovascular abnormalities were the most common concomitant disease (9.64%), followed by endocrine (4.62%), respiratory (3.12%), and renal (2.8%). Only 27 patients had minor surgery, accounting for 10.93% of the cases. Table 1.

**Table 1:** Characteristics of the study population and grade of surgery

Characteristics	Category	*N* (%)
Age	16 - ≤ 40	107(43.32)
	40 - ≤ 60	100(40.48)
	60 - ≤ 80	40(16.19)
Gender	Male	132(53.44)
	Female	115(46.60)
ASA status	I	229(92.71)
	II	14(5.66)
	III	4(1.62)
Main organ dysfunction	Cardiovascular	9(3.64)
	Respiratory system	3(1.21)
	Endocrine system	4(1.62)
	Renal system	2(0.81)
Grade of surgery	Minor	27(10.93)
	Intermediate	63(25.51)
	Major/complex	157(63.56)

The majority of patients (132) had general and urological surgery, while the remainder received orthopedic (70) and gynecological (45) procedures. During the data collection period, thyroidectomy (44), vaginal hysterectomy [22], and incision and drainage [18] were the most common operations performed in general surgery, gynecology, and orthopedics departments, respectively. Table 2: Full blood count (FBC) and echocardiography (Echo) were the most common 240 (97.16) and infrequent 4 (1.62%) tests among 350 investigations requested on all 247 individuals, respectively. Table 3.

**Table 2:** Types of surgery done during the data collection period

Types of operation		Number of operation
Gynecological	Trans vaginal hysterectomy	28
	Trans abdominal hysterectomy	9
	Suspension	2
	Myomectomy	6
	Total	45
Orthopedics	ORIF	7
	External Fixation	14
	Plate	3
	Contracture release	5
	Nailing	4
	FB removal	2
	Pinning	3
	Pin removal	2
	Incision and drainage	19
	Skin graft	6
	Amputation	5
	Total	70
General and urological surgery	Thyroidectomy	44
	Prostatectomy	19
	Mastectomy	4
	Fistulectomy	20
	Hydrocelectomy	4
	Excision	3
	Ligation	8
	Hernioraphy	12
	Colostomy closure	7
	Cholecystectomy	9
	Hemoroidectomy	2
	Total	132

**Table 3:** The number of investigations performed in comparison to those recommended by NICE

Type of surgery	ASA Status	*N*	Number of investigations performed/number of investigations offered according to the NICE recommendation								
			FBC	hemostasis	KFT	HbA1c	ECG	Echo	LFT	CXR	Urine test
Minor	I II III	26 1 0	20/9 1/1 0/0	0/0 0/0 0/0	0/0 1/1 0/0	0/0 0/1 0/0	0/0 0/1 0/0	0/0 0/0 0/0	0/0 1/1 0/0	0/0 0/0 0/0	3/0 0/0 0/0
Intermediate	I II III	53 8 2	52/37 8/5 2/2	2/3 1/2 0/1	2/0 4/4 2/2	0/0 0/0 0/0	6/4 4/6 1/2	0/0 1/2 1/1	2/0 4/4 2/2	1/0 1/2 0/1	0/0 4/4 0/2
Major/complex	I II III	150 5 2	150/134 5/5 2/2	3/2 2/3 1/1	15/19 2/3 1/1	0/0 0/2 0/1	11/9 2/3 1/1	1/2 0/1 1/1	15/8 2/3 1/1	0/1 2/2 1/1	8/7 1/3 1/1

When the type of surgery and ASA classification are taken into consideration for each component of the test requests, for example, out of 19 FBC tests requested by ASA 1 patients who had minor surgery, only 9 FBC tests were deemed appropriate based on NICE recommendations. Additionally, despite being indicated to have them prior to the procedure, none of the HA1cs were requested for the two ASA 2 patients who underwent major surgery. Table 3.

**Fig. 1:**
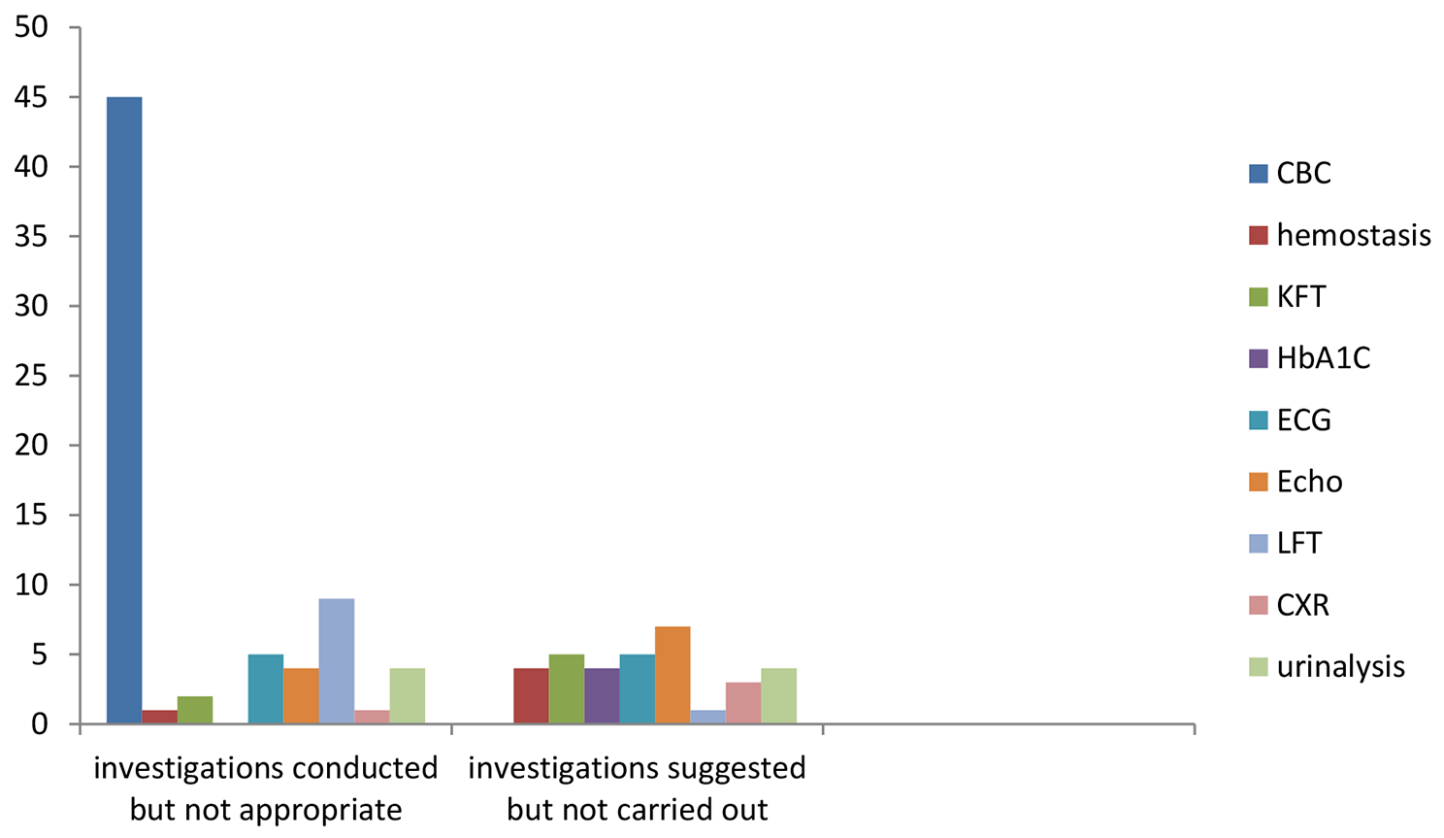
The number of investigations that were conducted inappropriately indicated investigations that were not completed in accordance with NICE recommendations

Overall, contrary to NICE recommendations, FBC was performed inappropriately as part of the preoperative investigation in 45 patients. In one patient without a clear indication, a hemostasis test was performed as part of the preoperative investigation. One patient was subjected to unnecessary chest x-rays. For 5 patients (ECG) and 4 patients (urinalysis), there was no clear indication. On the other hand, despite being indicated by NICE, PT/PTT in 4 and HbA1c in 4 patients were not performed. Figure 1.

**Fig. 2:**
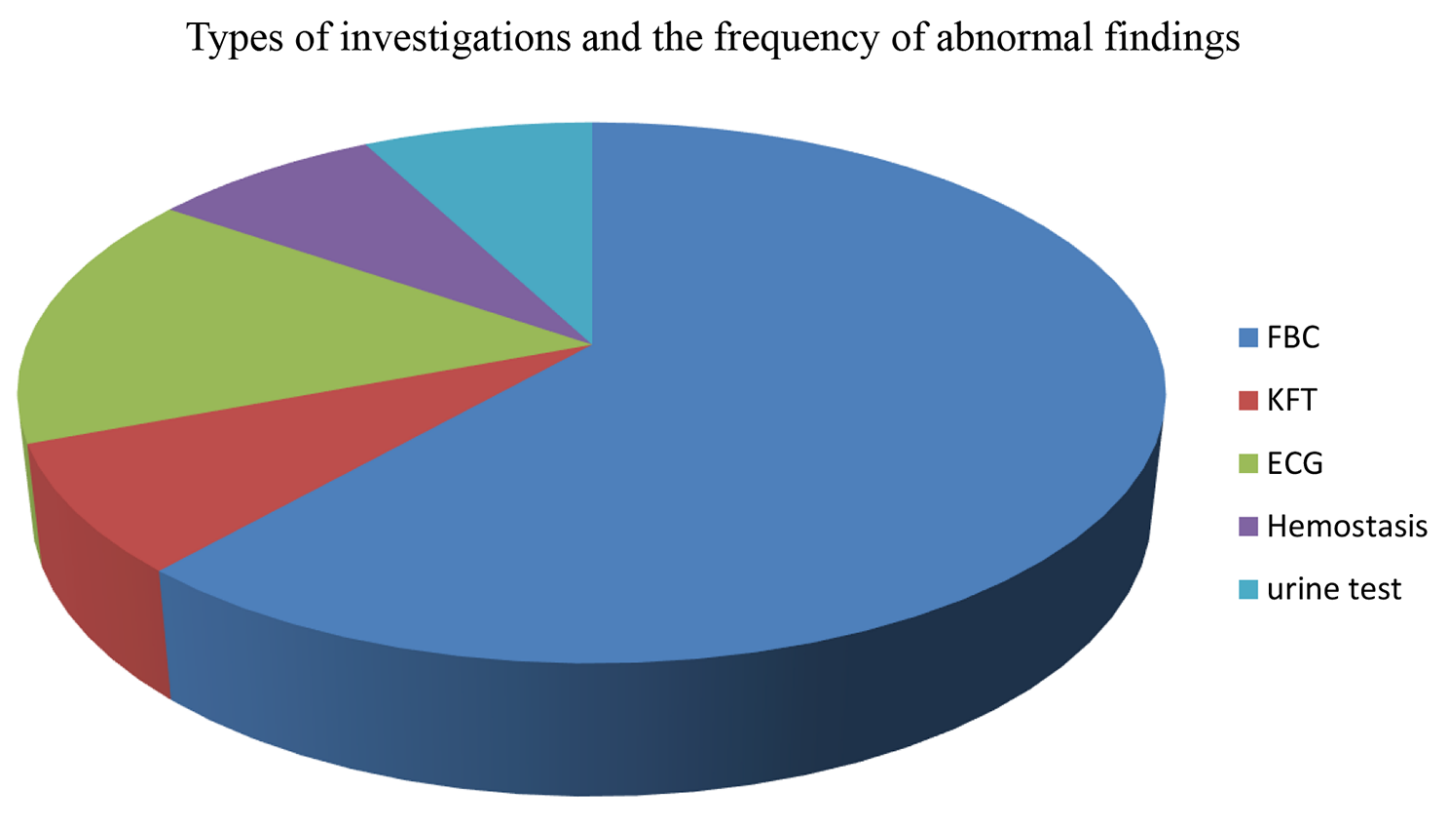
Types of investigations and the frequency of abnormal findings

Moreover, among the investigated laboratory and imaging workups, 8 FBC, 2 ECG, 1 KFT, 1 hemostasis, and 1 urine test showed abnormal results. In addition, the finding revealed that there was no patient whose investigations were conducted without a clear indication and whose results were found to be abnormal. Figure 2.

## Discussion

In this study, out of all the patients audited, a total of 350 investigations were requested. Among these, approximately 79.71% of the requested investigations were conducted following national guidelines. This aligns with findings from Sri Lanka, where adherence to national guidelines exceeded 75% [2]. Conversely, 71 tests (20.28%) were requested without proper justification, violating NICE guidelines. Research by Benarroch-Gampel et al. revealed that as many as 90% of patients underwent at least one preoperative test that wasn’t medically necessary [23]. The discrepancy in findings between their study and ours might be due to their use of different criteria for comparison and their retrospective review of patient records.

Our study’s results reveal that the FBC (240) test is the most frequently requested test, which differs from findings in a related study conducted in Sri Lanka and Texas. In those studies, the most commonly performed tests were ECG and hematological investigations, respectively [23, 24]. The variation in findings may be due to differences in the study’s design, sample size, and the types of surgeries included. For example, the initial audit involved 123 individuals undergoing urologic surgery exclusively. This might be because urologic conditions are often associated with age-related comorbidities, particularly cardiovascular disease [25, 26].

During the assessment of each investigation component for adherence to guidelines, it was observed that FBC was requested for nearly all patients as part of routine preoperative investigation before surgery (240 patients, 97.16%). Among these, 45 patients underwent unnecessary FBC requests, contrary to NICE guidelines. This proportion is notably higher than the findings of a previous study conducted in Sri Lanka, where 24 patients were subjected to unwarranted preoperative requests for complete blood count tests [24, 27].

The difference in findings could be due to the fact that the study by Abayasinghe Chamika et al. focused solely on the urology unit, potentially limiting variation in clinicians’ preoperative testing requests. However, despite this, the NICE guidelines advise against routinely requesting FBC for all patients before surgery. Instead, NICE recommended considering FBC for patients undergoing major or complex surgery, as well as for ASA3 and ASA4 patients undergoing intermediate surgery [13].

Regarding ECG, NICE recommends considering ECG requests for ASA 2 patients undergoing intermediate or major surgery, ASA 3 or 4 patients undergoing minor surgery without available ECG results from the previous year, and ASA 1 patients aged 65 or older scheduled for major surgery. However, contrary to these guidelines, preoperative ECGs were requested for five patients. In contrast, a study by P. Ranasinghe et al. found that non-recommended ECGs were requested in 30.6% of all patients [2].

The discrepancy between our results and theirs may be attributed to the study’s larger sample size (356), and the authors’ use of an outdated version of the NICE recommendation for comparison. Although ASA 3/4 patients with chronic liver disease, those taking anticoagulants or needing treatment regimen adjustments, and those necessitating clotting status assessment before intermediate or major surgery are recommended to undergo a hemostasis workup, PT/INR testing was conducted for 1 patient without evident justification.

Another notable discovery from this audit is the underutilization of certain tests recommended by the guidelines. For instance, despite NICE’s recommendation, hemostasis profile testing was omitted for four individuals. Similarly, kidney function tests were not conducted for 5 patients, and HbA1c tests were not performed for 4 patients, despite being indicated by the guideline. While routine testing in generally healthy individuals has been debated, scientific evidence also advocates for selective testing tailored to factors such as the patient’s medical status, the complexity of the planned surgery, and the likelihood of blood loss [13, 28]. Clinicians can confirm diagnoses, assess the severity and progression of diseases, and predict prognoses by utilizing the results of investigations performed on symptomatic and indicated patients [22].

### Limitation

The strength of this clinical audit is its potential to enhance patient safety and optimize healthcare delivery by identifying areas for improvement in preoperative investigation practice protocols. The first drawback of the audit was that it did not include who requested each test, as most patient cards do not record which practitioners requested particular investigations. The study’s second limitation is that it doesn’t look at whether patients who have abnormal results during or after surgery are given any special attention or therapy. Lastly, the study failed to evaluate the variables that lead to the over-ordering of preoperative investigations. Consequently, we advise that future investigators address the aforementioned constraints in their research on the ordering of preoperative examinations before surgery.

## Conclusion

In our hospital’s surgical clinical practice, preoperative investigations, notably FBC, liver function tests, and ECG, are still being excessively prescribed. On the contrary, we found that specific tests like HbA1c and echocardiography are necessary but have not been conducted yet. Avoiding routine testing does not mean avoiding preoperative investigation altogether. Instead, it involves following guidelines and carefully selecting which preoperative investigations to perform based on a comprehensive history, a physical examination, and the nature of the planned surgery.

### Recommendations

Based on the findings of the audit, the following suggestions are proposed:

1. Establish a clear, up-to-date, evidence-based national or institutional guideline for preoperative investigation protocols.
2. Ensure that this guideline is readily accessible at the location where preoperative investigation requests are made.
3. Regularly conduct audits to assess the effectiveness of these protocols in ensuring patient safety.

## Data Availability

No datasets were generated or analysed during the current study.

## Abbreviations

ASA: American Society of Anesthesiologists
CXR: chest x-ray
ECG: electrocardiography
Echo: echocardiography-resting
FBC: full blood count
HbA1c: Hemoglobin A1C
KFT: Kidney Function
LFT: lung function tests
NICE: National Institute for Healthcare Excellence
PI: preoperative investigation
